# Dynamics of transposable elements in recently diverged fungal pathogens: lineage-specific transposable element content and efficiency of genome defenses

**DOI:** 10.1093/g3journal/jkab068

**Published:** 2021-03-16

**Authors:** Cécile Lorrain, Alice Feurtey, Mareike Möller, Janine Haueisen, Eva Stukenbrock

**Affiliations:** 1 Environmental Genomics, Max Planck Institute for Evolutionary Biology, Plön 24306, Germany; 2 Environmental Genomics, Christian-Albrechts University of Kiel, Kiel 24118, Germany; 3 Université de Lorraine/INRAE, UMR 1136 Interactions Arbres/Microorganismes, INRAE Centre Grand Est—Nancy, Champenoux 54280, France

**Keywords:** transposable elements, effectors, genome architecture, *Zymoseptoria tritici*, repeat-induced point mutations, genome plasticity

## Abstract

Transposable elements (TEs) impact genome plasticity, architecture, and evolution in fungal plant pathogens. The wide range of TE content observed in fungal genomes reflects diverse efficacy of host-genome defense mechanisms that can counter-balance TE expansion and spread. Closely related species can harbor drastically different TE repertoires. The evolution of fungal effectors, which are crucial determinants of pathogenicity, has been linked to the activity of TEs in pathogen genomes. Here, we describe how TEs have shaped genome evolution of the fungal wheat pathogen *Zymoseptoria tritici* and four closely related species. We compared *de novo* TE annotations and repeat-induced point mutation signatures in 26 genomes from the *Zymoseptoria* species-complex. Then, we assessed the relative insertion ages of TEs using a comparative genomics approach. Finally, we explored the impact of TE insertions on genome architecture and plasticity. The 26 genomes of *Zymoseptoria* species reflect different TE dynamics with a majority of recent insertions. TEs associate with accessory genome compartments, with chromosomal rearrangements, with gene presence/absence variation, and with effectors in all *Zymoseptoria* species. We find that the extent of RIP-like signatures varies among *Z. tritici* genomes compared to genomes of the sister species. The detection of a reduction of RIP-like signatures and TE recent insertions in *Z. tritici* reflects ongoing but still moderate TE mobility.

## Introduction

Transposable elements (TEs), DNA elements that can replicate through transposition (*i.e.*, independently of the host DNA replication machinery) are ubiquitous in eukaryotic genomes. The TE content in fungal plant-pathogen genomes covers a wide range: from less than 1% to more than 90% of the genome in *Fusarium graminearum* and *Blumeria graminis*, respectively (Cuomo *et al.* 2007; Frantzeskakis *et al.* 2018). TEs are categorized into two classes, retrotransposons (class I) and DNA transposons (class II), based on their mechanism of transposition (Wicker *et al.* 2007). Retrotransposons replicate using an RNA intermediate to insert at a new position and DNA transposons replicate either by a mechanism of direct excision from double-stranded DNA (subclass I) or using single-strand excision followed by a rolling-circle mechanism (subclass II) (Wicker *et al.* 2007). TE classes are divided into orders that contain various numbers of superfamilies and families, which are categorized by coding sequence structure (Wicker *et al.* 2007). TEs can be autonomous (*e.g.*, LTRs and TIRs) or nonautonomous (*e.g.*, SINEs and MITEs). The latter relies on the replication machinery of autonomous TEs to transpose (Wicker *et al.* 2007). TE activity (*i.e.*, transposition) is known to have an overall negative impact on host fitness (Horváth *et al.* 2017). As a result, TEs engage in a co-evolutionary arms race dynamic with the host genome (Biémont 2010; Castanera *et al.* 2016).

Fungal genomes have evolved diverse genome defense mechanisms to regulate TE expansions. In addition to histone modifications and DNA methylation (Deniz *et al.* 2019), a fungal-specific mechanism called repeat-induced point (RIP) mutation specifically mutates duplicated sequences such as TEs (Gladyshev 2017). The underlying molecular mechanism of RIP is not clearly known for a vast majority of fungal species. However, a strong association of RIP and DNA methylations (particularly C5-cystosine methylation) of repeated sequences have been demonstrated in several species (Gladyshev 2017). In fungi, two main enzymes are known to be involved in RIP and DNA methylation: RID and DIM-2. However, these enzymes involved in RIP are not present in many fungal lineages suggesting a heterogeneous distribution of the RIP mechanism throughout the fungal kingdom (Bewick *et al.* 2019). RIP induces a dinucleotide bias in duplicated sequences by mutating G: C into A: T; this bias can be measured in fungal genomes, and quantified as a RIP signature (Gladyshev and Kleckner 2014, 2017). In “RIPed” genomes, RIP-induced mutations can result in large RIP affected regions (LRARs) that are large genomic regions consecutively affected by RIP (van Wyk *et al.* 2019). Genomes of several fungal pathogens have relatively high TE contents, while simultaneously exhibiting signatures of RIP (Gao *et al.* 2011; Gioti *et al.* 2013; Grandaubert *et al.* 2014; Dhillon *et al.* 2014; Fokkens *et al.* 2018). It remains unclear how TEs can maintain stable proportions in their host genomes with defense mechanisms such as RIP. It is worth to note that the maintenance of RIP is costly for the host and that RIP can be lost (Galagan and Selker 2004). In the genus *Neurospora*, closely related species show variation in RIP signatures, and *Neurospora* species with reduced RIP signatures exhibit TE expansions (Gioti *et al.* 2013). This dynamic between TE expansions and host defense mechanisms is summarized as the burst and decay model of TE evolution (Arkhipova 2018). This model assumes that TEs are active (*i.e.*, they burst in transposition or expansion) until they are inactivated by host defense mechanisms such as RIP (*i.e.*, they decay). As a consequence of the RIP-induced overaccumulation of mutations in TEs, dating specific TE families’ invasions is challenging (Grandaubert *et al.* 2014).

Recent population re-sequencing and functional studies have demonstrated that some TEs also play a major role for genome evolution (Dubin *et al.* 2018). TE insertions and how the host genome manages TE activity define genome architecture. Indeed, TEs can increase genomic plasticity by promoting chromosomal rearrangements and compartmentalization, duplicating or deleting genes, and altering gene expression. Often, the TE content is linked to compartmentalization of the genome with TE-enriched genomic compartments or regions associating with specific epigenetic signatures and changes in GC content (Duplessis *et al.* 2011; Bertazzoni *et al.* 2018; Chen *et al.* 2018; Frantzeskakis *et al.* 2018; Stam *et al.* 2018). Genome compartmentalization into TE-rich and TE-poor regions can sometimes be observed at the chromosomal level. Some fungal species harbor dispensable or accessory chromosomes, which contain a higher proportion of TEs than the core chromosomes as observed in the wheat pathogen *Zymoseptoria tritici* (Croll and McDonald 2012). Once considered as “junk DNA,” TEs have been shown in recent years to participate in adaptation to environmental changes of their hosts. For instance, in some plant-associated fungi, recent selective sweeps support evidence of TE-driven adaptation (Oggenfuss *et al.* 2020). TEs are also physically associated with pathogenicity-related genes (*i.e.*, effectors) of various fungal pathogen species, suggesting a role in effector gene diversification (Rouxel *et al.* 2011; Grandaubert *et al.* 2014; Soyer *et al.* 2014; Dong *et al.* 2015; Fokkens *et al.* 2018; Fouché *et al.* 2018). Thereby, TEs can mediate mutational changes with benefits to the host organism. TEs can even be co-opted, or domesticated by the host, and evolved to have a new function in the host. For instance, the effector AvrK1 in *B. graminis* f.sp. *hordei* is directly derived from a LINE retrotransposon (Amselem *et al.* 2015a). In *Z. tritici*, LTR retrotransposon insertion upstream of a multidrug efflux transporter has conferred fungicide resistance (Omrane *et al.* 2017). Multiple studies demonstrate the importance of TEs for fungal pathogenicity, yet little is known about the extent to which TEs function as key players in the global evolution of the genomes of fungal pathogens.

The wheat pathogen *Z. tritici* has emerged as a model to study fungal genome and TE evolution. Closely related species of *Z. tritici* have been isolated from wild grasses and barley, and include *Zymoseptoria passerinii*, *Zymoseptoria ardabiliae*, *Zymoseptoria brevis*, and *Zymoseptoria pseudotritici* (Stukenbrock *et al.* 2007, 2012). The genomes of the five species comprise conserved core chromosomes and variable accessory chromosomes which show low-gene density, low-transcriptional activity, and enrichment of the heterochromatin-associated histone mark H3K27me3 (Goodwin *et al.* 2011; Kellner *et al.* 2014; Schotanus *et al.* 2015; Feurtey *et al.* 2020). TEs have been investigated in the economically important wheat pathogen *Z. tritici*, describing for example the occurrence of RIP signatures in this species through the quantification of C to T transition mutations for the reference isolate IPO323 (Dhillon *et al.* 2014). Several associations have been made between TEs and *Z. tritici* virulence and fungicide resistance. *Z. tritici* genes encoding effectors and transporters involved in fungicide resistance physically associate with TEs (Omrane *et al.* 2017; Hartmann *et al.* 2018; Oggenfuss *et al.* 2020). Recent studies demonstrated that TEs in *Z. tritici* are de-repressed during the early stages of wheat infection (Fouché *et al.* 2020). Interestingly, the TE content varies between closely related *Zymoseptoria* species (Grandaubert *et al.* 2015). TEs in *Z. tritici* accumulate in recently founded populations outside of the center of origin due to relaxed purifying selection (Badet *et al.* 2020; Fouché *et al.* 2020; Oggenfuss *et al.* 2020). Despite these recent studies, little is yet known about how TEs and genome defense mechanisms have co-evolved and shaped host genomes over larger time scales within the *Zymoseptoria* genus.

In this study, we explore TE dynamics in 26 *Zymoseptoria* genomes, including 22 *Z. tritici* genomes and one genome from each of the four closely related species, *Z. passerinii*, *Z. ardabiliae*, *Z. brevis*, and *Z. pseudotritici*. We specifically addressed the following questions: (i) How do TE distributions and insertion ages impact genome architecture and plasticity? (ii) Does TE content correlate with gene presence/absence variation among genomes? (iii) What is the extent of variation in RIP among genomes? To answer these questions, we annotated the TE content of each of the 26 genomes *de novo* and analyzed TE landscapes within and among genomes. We found evidence for variation in the efficiency of RIP among *Z. tritici* genomes and among the different species genomes.

## Materials and methods

### Genomic and gene expression data

**Figure 1:**
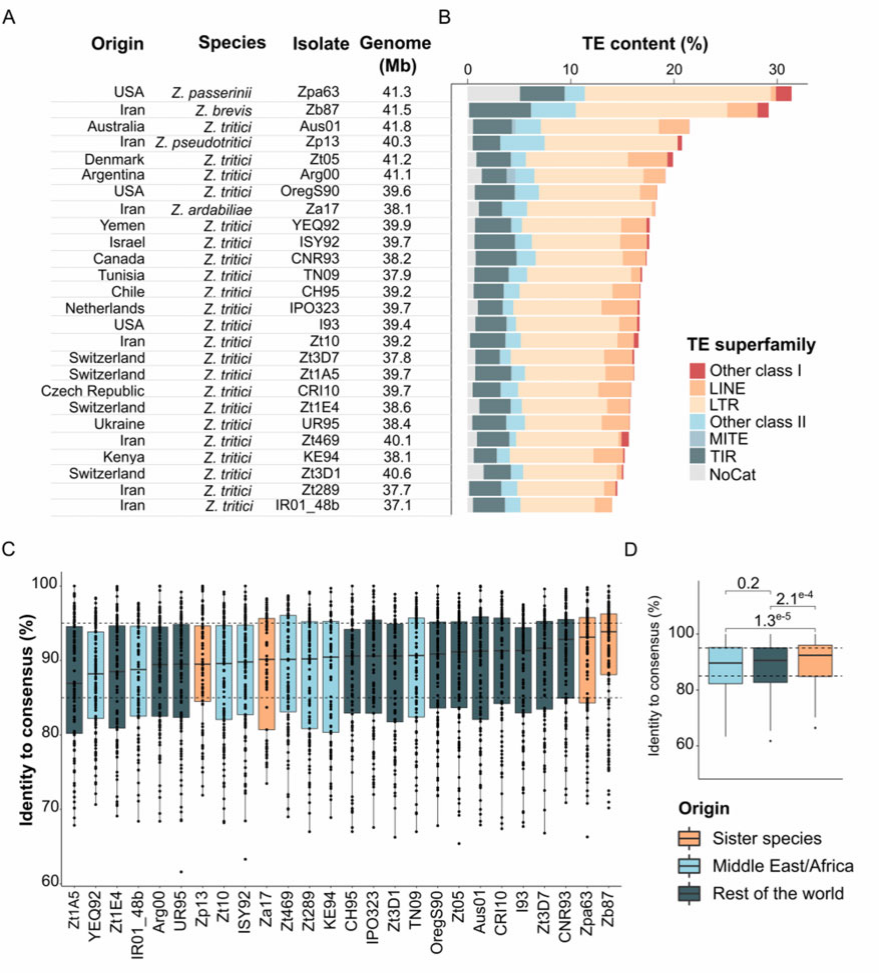
Transposable element content and identity variation in the *Zymoseptoria* genus. (A) Summary of the 26 long-read genome assemblies obtained from a worldwide collection of *Zymoseptoria* isolates, including the five species *Z. ardabiliae*, *Z. brevis, Z. pseudotritici*, *Z. passerinii*, and *Z. tritici*. Genome sizes are given in Mb. (B) Bars represent TE content (%) per genome estimated after REPET (Flutre *et al.* 2011) annotation. Colors represent TE order coverage with retrotransposons (LTR, LINE, and other class I orders in warm colors) and DNA transposons (TIR, MITE, and other class II orders in cold colors). Sequence identity distribution between TE copies and the respective consensus sequences (C) per genome and (D) per species and geographical origin. Each dot represents the median sequence identity of TE cluster. Boxplots are colored in regards to the species and isolate geographical origin.

All *Zymoseptoria* spp. isolates used in this study were obtained from publicly available long-read sequencing-based genome assemblies. In total, we included 26 high-quality assemblies of *Zymoseptoria* spp. genomes from PacBio sequencing (Badet *et al.* 2020; Feurtey *et al.* 2020; Möller *et al.* 2020). These include the genomes of *Z. ardabiliae* Za17, *Z. brevis* Zb87; *Z. passerinii* Zpa63, and *Z. pseudotritici* Zp13 (*et al.* 2020). We gathered a total of 22 *Z. tritici* genomes from strains isolated all over the world (Figure 1A) including eight isolates from Europe (the reference isolate IPO323 from the Netherlands); the four isolates Zt1A5, Zt1E4, Zt3D1, Zt3D7 (Plissonneau *et al.* 2018) from Switzerland, Zt05 from Denmark (Grandaubert *et al.* 2015), CRI10 from Czech Republic and UR95 from Ukraine (Badet *et al.* 2020); five isolates from America (CNR93 from Canada; I93, OregS90 from the USA; CH95 from Chile and Arg00 from Argentina) (Badet *et al.* 2020); one isolate from Oceania (Aus01 from Australia) (Badet *et al.* 2020); four isolates from Africa and the Middle-East (ISY92 from Israel; TN09 from Tunisia; KE94 from Kenya and YEQ92 from Yemen) (Badet *et al.* 2020); four isolates sampled in Iran (Zt10; Zt289; IR01_48b and Zt469) (Badet *et al.* 2020; Möller *et al.* 2020).

Gene expression during wheat infection (Haueisen *et al.* 2019), updated expression profiles on the last versions of genome assemblies and new gene predictions of the three *Z. tritici* isolates IPO323, Zt05, and Zt10, were used as previously described (Feurtey *et al*. 2020). In summary, RNAseq was performed during wheat infection time-course using strand-specific RNA-libraries from Illumina HISeq2500 sequencing, with 100 pb single-end reads. A total of 89.5 to 147.5 million reads per sample were obtained (Haueisen *et al.* 2019). To simplify the expression data, we combined all time-points from wheat infection and calculated expression levels in transcripts per million (TPM) as described earlier (Feurtey *et al*. 2020).

Prediction of the “secretome” and effector repertoire were performed as follows: (1) secreted proteins were predicted using SignalP4.1 with “sensitive” parameter settings (Petersen *et al.* 2011), (2) predicted secreted proteins were extracted from the proteome and effectors were predicted using EffectorP2.0 (Sperschneider *et al.* 2018) for each of the 26 *Zymoseptoria* genomes.

### Annotation of repeated elements and relative age of insertion

We used the REPET pipeline (https://urgi.versailles.inra.fr/Tools/REPET; Quesneville *et al.* 2005; Flutre *et al.* 2011) to annotate the repeat regions of *Z. ardabiliae* Za17, *Z. brevis* Zb87, *Z. pseudotritici* Zp13, *Z. passerinii* Zpa63, and the 22 *Z. tritici* isolates as described in (Feurtey *et al*. 2020). Briefly, we identified repeats in each genome by building TE consensus sequences as a proxy for the TE ancestral sequence. Each consensus is derived from multiple alignments of TEs in clusters. We then mined each *Zymoseptoria* spp. genome using the constructed TE consensus library to recover TE copies belonging to the same consensus. TE annotation metrics are summarized in Supplementary Table S1.

We assessed relative ages of TE insertions in Zymoseptoria spp. using the REPET similarity-based approach to measure the distributions of TE clusters’ sequence identities. Based on the concept of burst and decay evolution of TEs (Roessler *et al.* 2018), we analyzed sequence divergence of individual element clusters to assess the relative age of TE insertions in each genome (*i.e.*, TE spread in host genome). To the end, we assume that RIP-induced mutations has been is constant over time each genome. Based on this assumption, the extent of sequence similarity is proportional to the divergence time of copies. It is thereby possible to compare relative insertion ages of TE insertions within genomes (Supplementary Figure S1). We assessed sequence similarity within each TE cluster by comparing each TE copy to the consensus sequence.

### Analysis of transposon genomic environments

To further investigate intra-specific variation in TE content, we conducted a detailed comparison of the chromosomes of the 22 *Z. tritici* isolates. We first assessed the TE coverage per chromosome for each genome to detect intra-specific variation illustrating recent TE insertions. We quantified TE impacts on intra-specific small-scale chromosomal rearrangements in the 22 isolates of *Z. tritici*. Therefore, we used the software SyRi (Goel *et al.* 2019) and identified synteny breaks of a minimum of 500 bp. We assessed the distances from these synteny breaks to the closest transposons using bedtools “closest” function (Quinlan and Hall 2010). We extracted the TEs that overlapped with syntenic regions (*i.e., syntenic TEs*) in pairwise comparison with the reference genome of IPO323 using bedtools “intersect” function. We considered a TE insertion syntenic if the sequence has overlap of at least 50% with a syntenic region. Otherwise, the insertion is considered as *nonsyntenic*.

To illustrate the distribution of TEs along the chromosomes of *Z. tritici* isolates, we generated a Circos plot (Krzywinski *et al.* 2009). To this end, we counted TE in 100 kbp windows using bedtools “makewindows.” We fixed window coordinates based on the reference genome of IPO323 using orthologous genes. The genes located most closely to the borders were extracted from the reference genome of IPO323 using the bedtools “closest” function (Quinlan and Hall 2010). Orthologs of these genes were then extracted using the program Proteinortho (Lechner *et al.* 2011). The number of transposons per window was calculated using bedtools “intersect” function (Quinlan and Hall 2010).

To assess associations between the vicinity of TEs and genes potentially involved in plant infection, we used the previous functional annotations of predicted effectors and orthologous genes of Za17, Zpa63, Zb87, and Zp13, and the three *Z. tritici* isolates IPO323, Zt05, and Zt10 (Feurtey *et al*. 2020). We annotated the genes with presence/absence variation (PAV) among the 26 *Zymoseptoria* species-complex genomes. For this, we used PoFF, an extension of the software Proteinortho which integrates data on conserved synteny to detect orthologous relationships (Lechner *et al.* 2011). We differentiated genes as follows: (1) showing PAV among all 26 genomes as *PAV genes*, (2) genes present in all 26 *Zymoseptoria* genomes as *Core genes* and (3) genes present in all 22 *Z. tritici* isolates as *Core Z. tritici genes*. We also included predicted genes with TE-like domains (*e.g.*, transposase) in the TE repertoires to avoid considering TEs as *PAV genes*. To statistically assess associations between the vicinity of transposons and gene categories, we used the R package regioneR (Gel *et al.* 2015). We used the function “meanDistance” to test whether specific gene categories are closer to transposons than expected for a random distribution. We performed the permutation test with the “randomizeRegion” function and 1000 permutations. Randomizations were performed per chromosome.

### Repeat-induced point mutation (RIP) analysis

To evaluate the extent of RIP-like signatures among the genomes and TEs sequences of the 26 genomes of *Zymoseptoria* spp., we combined overall dinucleotide composition and window-based RIP indices. The dinucleotide compositions of (1) whole-genome and (2) TE copies were assessed by counting dimer frequencies using the function “compseq” from EMBOSS suite (Rice *et al.* 2000). Because RIP signatures strongly correlates with dinucleotide bias with containing A and T, we compared the observed dinucleotide frequencies in TE and gene sequences to expected dinucleotide frequencies (Gladyshev and Kleckner 2017). The expected frequencies are calculated on the false assumption that every dinucleotide pair has an equal frequency (*i.e.*, 0.065). RIP-like signatures were calculated, and Large RIP-affected genomic regions were determined, using the RIPper software (https://github.com/TheRIPper-Fungi/TheRIPper/, van Wyk *et al.* 2019). Regions of more than 4000 bp that are consecutively affected by RIP are considered to be “large RIP affected genomic regions.” For genome-wide RIP index assessments, we used default parameters of 1000 bp windows with a 500 bp step size. RIP Composite index values were calculated as follows: (TpA/ApT) – (CpA + TpG/ApC + GpT). A region is affected by RIP when the Composite index is >0 (van Wyk *et al.* 2019). To calculate the RIP Composite index of each transposon copy, we used 50 bp nonoverlapping windows using a custom script available at 10.5281/zenodo.4322565.

## Data Availability

Supplementary materials are available at Figshare: https://doi.org/10.25387/g3.14134724. All data produced and analyzed in this study including TE annotations and consensus libraries are available at 10.5281/zenodo.4322565. The genome sequencing datasets analyzed in the current study are available in the NCBI Short Read Archive (https://www.ncbi.nlm.nih.gov/bioproject/?term=) under the BioProject accession numbers: PRJNA638605, PRJNA639021 (Zpa63), PRJNA638553 (Zb87), PRJNA638515 (Zp13), PRJNA638382 (Za17), PRJNA414407 (Zt05 and Zt10), PRJNA614493 (Zt289 and Zt469). Gene annotations from Feurtey *et al.* (2020) are available at 10.5281/zenodo.3820378. Genome assemblies and annotations of worldwide isolates (Arg00; Aus01; CH95; CNR93; CRI10; I93; IR01_48b; ISY92; KE94; OregS90; TN09; UR95; YEQ92; Zt1A5; Zt1E4; Zt3D1 and Zt3D7) were downloaded from the European Nucleotide Archive (http://www.ebi.ac.uk/ena) under the BioProject PRJEB33986. The genome sequence of the reference isolate IPO323 is available at: http://genome.jgi.doe.gov/Mycgr3/Mycgr3.home.html. In planta RNA‐seq datasets for *Z. tritici* IPO323, Zt05, Zt10 from (Haueisen *et al.* 2019) are available at the accession number NCBI Gene Expression Omnibus GSE106136 and *in vitro* RNA-seq data for Za17, Zp13, and Zb87 from Grandaubert *et al.* (2015) are available at the accession number NCBI BioProjects PRJNA277173, PRJNA277174, and PRJNA277175.

Supplementary material is available at https://doi.org/10.25387/g3.14134724.

## Results

### Transposable elements content varies in the genomes of the *Zymoseptoria* species-complex

Overall, the TE proportions of *Z. passerinii*, *Z. ardabiliae*, *Z. brevis*, and *Z. pseudotritici* genomes are higher than the TE proportions of *Z. tritici* isolates (Supplementary Table S1; Figure 1). Outside of *Z. tritici*, TE content ranges from 6.9 Mb in the *Z. ardabiliae* Za17 genome (18.2% of the genome) to 12.9 Mb in the *Z. passerinii* Zpa63 genome (31.4% of the genome; Figure 1B; Supplementary Table S1). Among *Z. tritici* isolates, TE content ranges from 5.50 Mb in the Iranian isolate IR01_48b genome (14.0% of the genome) to 8.79 Mb in the Australian isolate Aus01 genome (21.5% of the genome) (Figure 1B; Supplementary Table S1). Most of the TE coverage in all genomes of *Zymoseptoria* species consists of LTR-retrotransposons. LTRs elements represent 2.6–7.4 Mb of the genomes of *Z. tritici* (IR01_48b) and *Z. passerinii*, respectively. Among these LTRs, we only found *Copia* and *Gypsy* elements, except in the Iranian *Z. tritici* isolate Zt469, in which we also identified elements belonging to the unique *Bel-Pao* cluster family (representing 0.85 Mb; Supplementary Table S1). LINE elements are completely absent from the genome of *Z. pseudotritici* and comprise only 0.29% (0.1 Mb) of the genome of *Z. ardabiliae* and 0.5% (0.2 Mb) of the genome of *Z. passerinii*. In contrast, *Z. brevis* Zb87 appears as an outlier: its genome contains 1.2 Mb (2.9% of the genome) of LINE elements. Among *Z. tritici* isolates, LINE content ranges from 0.29 (in Zt469) to 3.83% (in Zt05). Such distinct patterns of LINE superfamily TEs suggest that independent expansions and/or losses of these rare elements occurred in the genomes of the *Zymoseptoria* genus. In addition, LINE elements are enriched on accessory chromosomes and their different enrichment may reflect presence/absence variation of accessory chromosomes. Other elements such as TIR and LTR are also enriched on accessory chromosomes (Supplementary Figure S2).

### Many recent TE insertions postdate diversification of the *Zymoseptoria* genus

Sequence similarities between TEs and their cognate consensus sequences in the genomes of *Zymoseptoria* spp. range from 65.4 to 100% sequence identity (Figure 1C). Based on the approach used in Maumus and Quesneville (2014), we defined TE categories based on thresholds as follows: (1) copies with less than 85% sequence identity to the consensus comprise *old* insertions and (2) copies with 85 to 95% sequence identity are *intermediate* insertions and (3) copies with more than 95% identity with the consensus sequence represent *recent* insertions. Based on these criteria, we show that *old* insertions comprise 20.3 (Aus01) to 37.8% (YEQ2) of the total TE repertoire (Figure 1C; Supplementary Figure S2D). However, these copies represent only 0.27 (Zp13–0.67%) to 0.76 Mb (YEQ2–1.9%) of the genome coverage, suggesting degeneration of these elements since their insertion. On the contrary, *recent* insertions comprise 28.6 (Zt3D1) to 39.6% (Zb87) of the total TE repertoire covering from 2.8 (IR01_48b—7.5% of the genome) to 7.46 Mb (Zpa63–18.0% of the genome) (Figure 1C; Supplementary Figure S2C). The majority of *recent* TE insertions are retrotransposons while the majority of *old* insertions are retrotransposons and DNA transposons (Supplementary Figure S2, B and C). Taken together these results suggest that at least a fraction of TEs has been recently active within genomes.

### Past and present TE activity has shaped the genomes of *Zymoseptoria* species

**Figure 2:**
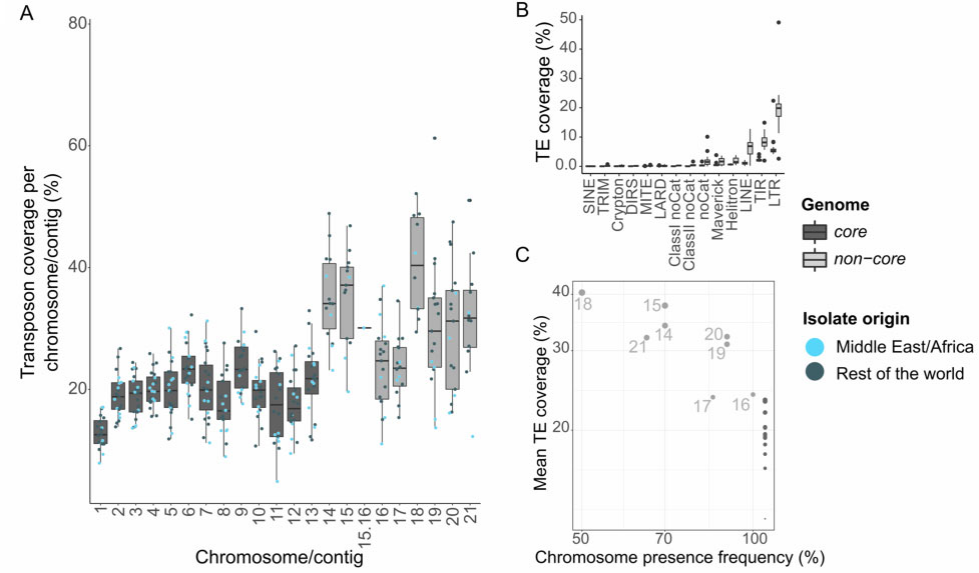
Transposable element insertions shape genome compartmentalization in *Z. tritici* genomes. (A) TE content variation of the core (dark gray) and accessory (light gray) chromosomes of *Z. tritici* genomes. The boxplots represent the distribution of TE coverage percentage per chromosome or contig among the 22 *Z. tritici* isolates. Jitters in the boxplot represent TE content per chromosome of individual isolates. Light blue: isolates from Middle East/Africa and dark blue: isolates from other worldwide locations. Chromosome 15.16 represents the chromosome fusion of the *Z. tritici* isolate YEQ92 from Yemen described in Badet *et al.* (2020). (B) Average coverage of distinct TE orders of core and accessory chromosomes of the 22 *Z. tritici* isolates. (C) Frequency of accessory chromosomes among the 22 *Z. tritici* isolates as a function of mean TE coverage.

TE-rich accessory chromosomes represent an ancestral trait of the genome architecture within the *Zymoseptoria* species-complex (Feurtey *et al*. 2020). In agreement with previous reports (Grandaubert *et al.* 2015), we found that transposons are enriched on accessory chromosomes (Figure 2A; Supplementary Figure S3). The proportion of TEs per core chromosome or contig varies from 5.7 (chr 11 of Zt469) to 33.3% (chr 9 of Zt05) (Figure 2A; Supplementary Figure S3). The proportion of TEs on accessory chromosomes or contigs varies from 11.0 (chr 16 of Zt469) to 61.2% (chr 20 of Aus01). Variation on the core chromosomes is mostly driven by LTR retrotransposons and TIR elements, which are the most abundant elements within whole genomes (Figure 2B). On accessory chromosomes, we observe more “rare elements” such as LINE elements (on average 2.24% coverage on core vs. 5.90% on accessory chromosomes), Maverick (on average 1.9% coverage on core *vs* 2.6% on accessory chromosomes), and Helitron (on average 1.2% coverage on core *vs* 1.94% on accessory chromosomes). This enrichment of rare elements on accessory chromosome contributes to the variation in TE coverage on accessory compared to TE coverage on core chromosomes (Figure 2B).

To investigate further the impact of TEs on genome organization, we identified *synteni*c and *nonsyntenic* regions using a whole-genome alignment approach in pairwise comparison for *Z. tritici* isolates to the reference IPO323 and further identified the TEs present in these two types of regions. We observed that a majority of TEs are found in nonsyntenic regions (from 60% of chr 9 in Zt469; 98% of chr 10 in Zt1E4) while only few insertions overlap in at least 50% of sequence length with syntenic regions (Supplementary Figure S4B). Finally, we observe that accessory chromosomes with a higher TE content are less abundant in the 22 *Z. tritici* isolates (Figure 2C). This could be due a lower rate of recombination on the chromosomes found at lower frequency, which could facilitate accumulation of TEs on these chromosomes (Stukenbrock and Dutheil 2018). Alternatively, a higher TE content could mean that maintaining the accessory—and thus potentially not essential—chromosome has a higher fitness cost and it is thus lost more readily.

In a previous study, we identified a large interspecies chromosomal inversion on contig_65 of *Z. brevis* Zb87 and contig_76 of *Z. pseudotritici* Zp13 compared to the chromosome 2 of *Z. tritici* IPO323 (Feurtey *et al.* 2020). This large rearranging region actually consists of several inversions between clusters of TEs (Supplementary Figure S5). We identified three TE clusters in Zb87 (46 TEs representing 0.12 Mb) and Zp13 (30 TEs representing 0.28 Mb) that surround the inverted loci and are absent in *Z. tritici* IPO323 (Supplementary Figure S5). We further scrutinized the link between TEs and rearrangements by pairwise comparisons of the *Z. tritici* isolates to the reference IPO323 (Supplementary Table S3). Of all inverted regions identified per isolate, 27% (Zt469) to 72% (Zt1A5) are flanked (separated by less than 10 bp) by transposons both upstream and downstream (Supplementary Table S3). In total, we counted from 15 (Zt10) to 52 (Zt1E4) intra-specific inversions comprising more than 500 bp in pairwise comparisons to the reference isolate IPO323 (Supplementary Table S3). The cumulative size of these inversions ranges from ∼136.7 to ∼845.2 kb in Zt1E4 and Zt469, respectively. From 10 (Zt469) to 128 (Zt05) genes are located in inverted genomic regions (Supplementary Table S3). One inverted region of 170 kb is found on chromosome 13 in the reference genome but absent from the other isolates. Taken together these results indicate that TEs have facilitated genome rearrangements and have shaped genome compartmentalization in *Zymoseptoria* species.

### Presence/absence variation and effector genes associate with transposable elements

**Figure 3:**
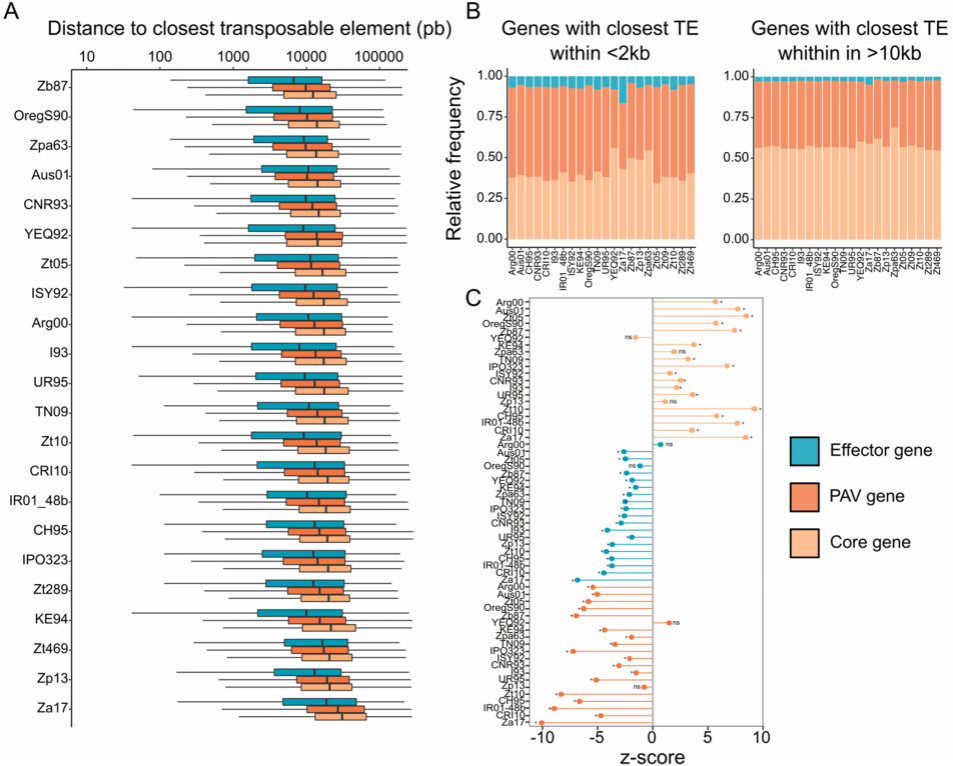
Transposable element insertions impact on gene content of *Zymoseptoria* spp. (A) Distribution of distance between effectors (blue), genes with presence-absence polymorphisms (PAV) among the 26 genomes of *Zymoseptoria* spp. (dark orange) genes and core genes (light orange) to the closest TE per chromosome/contig. (B) Relative frequency of predicted effectors, *PAV* and *core* genes found within the vicinity of TEs (<2 kb distance) and distant from TEs (>10 kb distance). (C) Z-scores obtained from permutation tests illustrate the strength of the association tested. Z-scores are defined as the distance between the expected value and the observed one, measured in standard deviations. Negative z-scores describe distances to TEs shorter than expected by chance while positive scores describe larger distance to TEs than expected by chance. Significant *P*s <0.05 of 1000 iterations permutation tests are indicated by *.

To assess the potential association of TEs with the high gene presence/absence variation described in *Z. tritici* (Plissonneau *et al.* 2018; Badet *et al.* 2019), we explored the distances between genes and TEs. Permutation tests were performed to compare the distributions of predicted effector, *PAV* and core genes along the genomes of the 26 *Zymoseptoria* spp. We obtained Z-scores to evaluate the strength of associations between TEs and genes (Figure 3C; Supplementary Table S4). We found that genes showing *PAV* and predicted effector genes are significantly closer to TEs in the genomes of 23 out of the 26 *Zymoseptoria* spp. genomes (permutation test of 1000 iterations, *P* < 0.05) with a median distance per chromosome ranging from 5.9 (*Z. brevis* Zb87) to 18.8 kb (*Z. ardabiliae* Za17) for predicted effector genes and from 9.3 kb (*Z. tritici* OregS90) to 26.6 kb (*Z. ardabiliae* Za17) for *PAV* genes (permutation test of 1000 iterations, *P* < 0.05; Figure 3, A and C).On the contrary, core genes are found to be significantly more distant to TEs with a median distance per chromosome ranging from 12.1 (*Z. brevis* Zb87) to 30.7 kb (*Z. ardabiliae* Za17; permutation test of 1000 iterations, *P* < 0.05; Figure 3, A and C). In addition, the proportion of genes found in the vicinity of TEs (within a distance of <2 kb flanking region) is enriched in predicted effector and *PAV* genes (Figure 3B).

To assess if particular TE families are associated with genes, we tested the distribution of the major TE families from class I (*i.e.*, *Copia*, *Gypsy*, and *LINE* elements) and class II (*TIR*, *Helitron*, and *MITE* elements) (Supplementary Table S5). In all *Zymoseptoria* genomes *TIR* and *MITE* elements are located significantly closer to *PAV* genes than expected from a random distribution (applying a permutation test of 1000 iteration *P* < 0.05) (Supplementary Table S5). *Copia* elements are significantly closer to *PAV* genes than expected from a random distribution in *Z. tritici* (IPO323, CRI10, and Zt10), *Z. passerinii*, and *Z. ardabiliae*. In contrast, class I *Gypsy* elements are more distant or nonsignificantly associated to *PAV* genes than expected from a random distribution in all *Zymoseptoria* genomes even though they are the most abundant TEs (permutation test of 1000 iteration *P* < 0.05) (Supplementary Table S5). Effector genes associate with TEs in each genome, but not with the same elements. Effector genes of *Z. tritici* IPO323 associate with *TIR* elements while in Zt10 effector genes associate with *Helitron* and *Copia* elements but not *TIR* elements (Supplementary Table S5). In conclusion, different TE families are found closer to predicted effector and *PAV* genes within the genomes of the *Zymoseptoria* species-complex. This suggests that TEs could contribute to genetic diversity and potentially play a role in the evolution of pathogenicity-related genes in the *Zymoseptoria* species-complex (Badet *et al.* 2020; Oggenfuss *et al.* 2020).

### RIP-like signatures are reduced on TEs of *Z. tritici* isolates compared to other *Zymoseptoria* genomes

**Figure 4:**
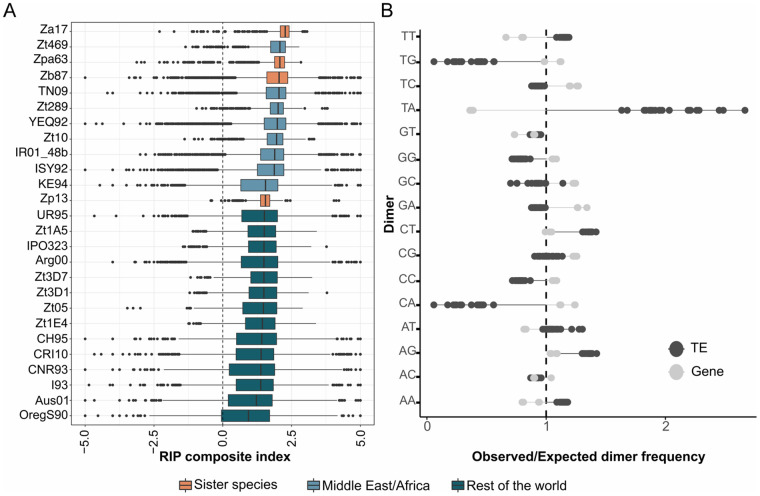
Signatures Repeat Induced Point mutations (RIP) in transposable elements of *Zymoseptoria* spp. Genomes. (A) Distribution of Composite RIP index (CRI) frequencies of TE sequences estimated using a 50bp sliding windows approach as follows: CRI = (TpA/ApT) – (CpA + TpG/ApC + GpT) for *Z. ardabiliae*, *Z. brevis*, *Z. pseuditritici*, and *Z. passerinii* (orange); for Iranian and African *Z. tritici* isolates (light blue) and for Z. tritici isolates from the rest of the world (dark blue). Vertical dash lines exhibit the threshold (0) above which CRI values indicate a RIP signature. (B) Dinucleotide frequencies in *Zymoseptoria* TE versus gene sequences. Lolipop plot represents the dinucleotide frequencies observed in TEs (dark gray) and genes (light gray) for all 26 *Zymoseptoria* genomes. Ratios of observed versus expected dinucleotide frequencies are reported. The Expected frequencies are calculated on the (false) assumption that every dimer has equal frequency.

We evaluated the RIP-like signatures in the *Zymoseptoria* genomes to estimate to what extent RIP affects different species of the genus. To this end, we scanned each genome and calculated RIP indices and dinucleotide frequencies (see Methods). In total, RIP-like signatures affect between 14.6% (in OregS90) and 34.5% (in Zpa63) of the total genomic content (Table 1). Composite index scan using 1kbp windows of the whole genome revealed that on average and as expected regarding TE content, accessory chromosomes are more RIPed than core chromosomes in all genomes (Table 1). Noteworthy, the presence/absence patterns of accessory chromosomes do not drive the changes in RIP signatures levels. Indeed, the isolates with the highest RIP-like signatures, such as Zt10 and IR01_48b, only carry five and four accessory chromosomes over the height accessory chromosome set. Among isolates with low RIP-like signatures, such as OregS90, I93 and Aus01 carry six, six and seven accessory chromosomes respectively (Badet *et al.* 2020)(Table 1). Overall, based on the estimation of RIP composite indices per 50 bp windows of TEs, the majority of TEs in *Zymoseptoria* exhibit RIP-like signatures (74–99% of TEs are RIPed), based on the estimation of RIP composite indices per 50 bp windows of TEs (Figure 4A). However, we find that RIP efficacy is not equivalent for all types of TEs. The small-sized and nonautonomous TEs such as MITE DNA-transposons are less affected by RIP (Supplementary Figure S5). The occurrence of RIP-like signatures on more recent TE copies suggests that the RIP machinery remains active in *Zymoseptoria* species. The large number of RIPed TEs suggests that TEs are mainly controlled by RIP-induced decay of TE copies in *Zymoseptoria*.

**Table 1:** Repeat-induced point (RIP) mutation signatures in the genomes of *Zymoseptoria* species

	Genome size (Mbp)	GC content of entire genome (%)	**Total estimated genome-wide RIP**^a^ **(%)**	Average size of LRAR (kbp)	**Average GC content of LRAR**^a^ **(%)**	Sum of all LRAR (Mbp)	Average composite index on core/ accessory chromosomes
*Z. ardabiliae* (Za17)	38.1	51.42	22.85	25.4	41.05	7.3	−0.18/0.52
*Z. brevis* (Zb87)	41.6	49.97	30.76	19.1	40.66	11.9	0.04/0.16
*Z. pseudotritici* (Zp13)	40.3	51.6	25.84	24.1	44.33	9.3	−0.20/0.13
*Z. passerinii* (Zpa63)	41.4	49.71	34.52	19.8	40.8	12.2	ND^a^
*Z. tritici* (Zt469)	42.9	48.52	17.44	18.2	41.81	7	−0.24/−0.13
*Z. tritici* (Zt289)	39	51.53	18.55	15.9	42.74	6.3	−0.28/0.18
*Z. tritici (Zt10)*	39.2	51.77	22.05	16.8	42.84	8.1	−0.25/0.27
*Z. tritici* (IPO323)	39.7	52.13	19.78	13	43.48	7.2	−0.36/−0.28
*Z. tritici* (Zt05)	41.2	51.94	21.77	13.5	43.64	8.2	−0.31/0.24
*Z. tritici* (Zt1E4)	38.7	52.26	16.65	10.4	43.57	5.6	−0.37/0.14
*Z. tritici* (Zt3D1)	40.6	52.01	19.55	11.7	43.64	7.1	−0.31/0.22
*Z. tritici* (Zt1A5)	39.7	52.2	17.41	11.1	43.59	6.2	−0.36/0.15
*Z. tritici* (Zt3D7)	37.9	52.22	17.4	11.7	43.39	5.9	−0.33/0.03
*Z. tritici* (Arg00)	41.1	51.87	20.03	12.3	43.71	7.3	−0.3/0.26
*Z. tritici* (Aus01)	41.7	52.07	18.61	12.2	44.48	6.8	−0.31/0.18
*Z. tritici* (CH95)	39.2	52.05	17.72	11.3	43.34	6.1	−0.32/0.10
*Z. tritici* (CNR93)	38.2	52.28	16.46	11.4	43.28	5.5	−0.33/0.34
*Z. tritici* (CRI10)	39.7	52.08	18.2	11.2	43.29	6.2	−0.32/0.11
*Z. tritici* (I93)	39.4	52.02	18.79	10.5	42.86	6.6	−0.31/0.13
*Z. tritici* (IR01_48b)	37.1	52.18	18.19	15.4	43.01	5.9	−0.29/−0.02
*Z. tritici* (ISY92)	39.7	51.75	21.56	16.6	42.89	7.9	−0.25/0.41
*Z. tritici* (KE94)	38.1	52.17	18.26	14.2	43.24	6.4	−0.32/0.08
*Z. tritici* (OregS90)	39.6	52.4	14.61	9.4	43.68	4.8	−0.36/0.03
*Z. tritici* (TN09)	37.9	51.74	22.06	16.8	43.06	7.5	−0.19/0.43
*Z. tritici* (UR95)	38.3	52.12	17.44	12.5	43.44	5.9	−0.34/0.16
*Z. tritici* (YEQ92)	39.8	51.54	23.72	18.0	42.65	8.0	−0.17/0.36

a ND: the genome assembly of *Z. passerinii* does not have a chromosome level assembly.

We observe however, a considerable difference in the efficacy of RIP among *Z. tritici* isolates. Median RIP composite index in TEs per species range from 0.93 (OregS90) to 2.26 (Za17). NonRIPed TE represents between 2.2 (Zt289) and 26.1% (OregS90) in the *Z. tritici* isolates (Figure 4A). We observe a decrease of RIP composite indices in *Z. tritici* isolates sampled from Europe, America, and Oceania compared to *Z. tritici* isolates sampled closer to the center of origin and in the closely related sister species (Figure 4A).

To further investigate this, we assessed the overall genome composition and TE sequences of the 26 genomes of *Zymoseptoria* as the presence of a strong bias in CpA dinucleotides is correlated to RIP signatures (Gladyshev and Kleckner 2017). To investigate potential over- or under-representation of dinucleotide containing A and T in TE sequences, we compared the dinucleotide frequencies in TEs to genes sequences of each isolate (Figure 4B). Overall, TE and genes dinucleotide composition clearly differ. In particular, we observed that TT, TA, CT, AT, AG, and AA dimers always have an observed frequency higher than expected in TEs and lower than expected in genes for all 26 *Zymoseptoria* genomes (Figure 4B; Supplementary Table S8). On the contrary, TG, TC, GG, GC, and CA have lower frequencies observed in TEs. In addition, the strongest bias of TA dinucleotides are found in TE sequences of the three sister species Za17 (ratio observed versus expected frequency of 2.63), Zpa63 (ratio observed versus expected frequency of2.45), and Zb87 (ratio observed versus expected frequency of2.42), right before the *Z. tritici* isolates from Middle East (Supplementary Table S8). On the other hand, TE sequences of *Z. tritici* isolates from Europe and America show a decrease in TA dinucleotides over-representation (Supplementary Table S8).

As RIP seems to affect TEs differently, this reduction of RIP can be associated with the variation in TE repertoires among the genomes of *Z. tritici* isolates. We observed that the proportion of MITE elements (*i.e.*, elements that are less affected by RIP) does not correlate with this reduction of RIP in TE copies (Supplementary Figure S6). The Iranian *Z. tritici* isolates comprise between 29 to 121 MITE copies while the other isolates comprise 68 to 156 MITE copies (Supplementary Table S1).

In addition, the average size of LRARs can be used as a proxy for RIP mechanism efficiency because it reflects to which extent a highly repeated region is affected by RIP. In the genomes of *Zymoseptoria* species, LRARs have an average size between 9.4 kb (in OregS90) and 25.4 kb (in Za17) and comprise large AT-rich regions (Table 1). LRAR average sizes in the *Z. tritici* isolates from Europe, America, and Australia are reduced compared to the other members of the *Zymoseptoria* species-complex and *Z. tritici* isolates coming from the Middle East and Africa (Table 1). It is worth noting that *Z. tritici* genomes with lower LRARs average size do not have significantly lower TE contents compared to the Iranian isolates. This indicates that the average size of LRARs is a good indicator for RIP efficiency. Besides TE sequences, only a small number of genes exhibits RIP signatures, including 43 genes in *Z. pseudotritici* and 92 genes in *Z. tritici* Zt05 (Supplementary Table S7). We conclude that RIP is highly efficient to inactivate TEs in the genomes of *Zymoseptoria* species but shows evidence of a lower efficacy in the genomes of the isolates of *Z. tritici* outside of center of origin.

## Discussion

Growing evidence demonstrates that TEs represent key players for the evolution and adaptation of fungal plant pathogens (Möller and Stukenbrock 2017). We investigated how past and present transposition events have shaped genome evolution of a major wheat pathogen and its wild-grass infecting sister-species. Within the genus *Zymoseptoria*, TEs associate with effector genes and PAV genes, as previously described for isolates of *Z. tritici* (Plissonneau *et al.* 2018). This suggests that TEs are either directly or indirectly involved in evolution of effectors and PAV genes in *Zymoseptoria* species as demonstrated in other species (Faino *et al.* 2016). TEs may therefore represent a major driver of *Zymoseptoria* spp. genome evolution.

Our detailed transposon analysis confirmed a conserved genome compartmentalization of TEs in accessory regions among species of the *Zymoseptoria* genus. TE accumulation is often associated with genome size expansions (Raffaele and Kamoun 2012). Here, we did not observe a clear correlation between genome size expansion and TE content. However, we did observe that genomes higher than 40 Mb contain also high TE coverage. A genome compartmentalization involving accessory chromosomes with highly dynamic TE regions was first demonstrated in the IPO323 reference genome of *Z. tritici* (Goodwin *et al.* 2011; Feurtey *et al.* 2020). Here, we show that his type of genome compartmentalization is an ancestral trait among *Zymoseptoria* species. The impact of purifying selection on the more gene-dense core chromosomes is known to be considerably stronger compared to the transposon-dense accessory chromosomes (Grandaubert *et al.* 2019). It is possible this relaxation selective constraints on accessory chromosomes allows TEs to accumulate in these regions. This effect may be emphasized by the lower recombination rate on the accessory chromosomes (Stukenbrock and Dutheil 2018).

TEs are involved either directly or indirectly in the genome plasticity of fungi (Faino *et al.* 2016). In this study, we explored the impact of TEs on genome architecture and gene presence/absence variation between and within species of *Zymoseptoria*. We observed slightly different TE repertoires at the superfamily level among the *Zymoseptoria* spp. genomes. The main difference resides in the proportion of rare elements such as LINEs. We observed that these elements are completely absent from *Z. pseudotritici* while they are abundant in higher proportions in *Z. brevis* and *Z. tritici* genomes. We hypothesize that the latter increase results from independent expansions of LINE elements in these two species. An alternative hypothesis is that the differences in LINE content could be due to different efficiencies in silencing these elements. This would however imply different mechanisms of LINE control in *Z. ardabiliae*, *Z. pseudotritici*, and *Z. passerinii* which are not present in *Z. brevis* and *Z. tritici*. Purifying selection against TE insertions has been demonstrated in *Z. tritici*, however, it was not shown to target specific TE superfamilies rather than others (Oggenfuss *et al.* 2020). Furthermore, the LINE copies in *Z. tritici* and *Z. brevis* do not exhibit particularly increased RIP signatures compared to other TEs.

TEs associate with regions exhibiting inter- and intra-species chromosomal rearrangements. Furthermore, we found that genes affected by presence/absence variation between and within species are located close to TEs. Such association of gene presence/absence with TEs has also been observed in other plant pathogenic fungi such as the rice blast *M. oryzae* (Yoshida *et al.* 2016; Bao *et al.* 2017). In addition, we demonstrate that among the 26 genome assemblies investigated here, PAV genes specifically associate with MITE and TIR elements. We found these elements to be less abundant in the genomes but also to be older compared to other elements such as *Copia* and *Gyspy* retrotransposons. These findings might suggest that some elements have been selected or linked to selected regions (typically near effector genes) in populations of *Z. tritici*.

TEs associate with effector genes in *Zymoseptoria* species, supporting the importance of TE-driven gene evolution for pathogenicity. In addition to the wheat pathogen *Pyrenophora tritici-repentis*, the pathogenicity-related protein ToxA is found in the two other wheat-associated pathogens, *Parastagonospora nodorum* and *Bipolaris sorokiniana* (McDonald *et al.* 2019)*.* Horizontal transfer of the ToxA encoding gene was demonstrated between these wheat-associated species and transfer was mediated by a DNA transposon from the hAT family (McDonald *et al.* 2019). TE insertions that facilitate variation of pathogenicity-related traits have been reported in several other fungal pathogens, including *L. maculans*, *F. oxysporum*, *M. oryzae*, and *Verticillium dahliae* (Rouxel *et al.* 2011; Amselem *et al.* 2015b; Faino *et al.* 2016; Fokkens *et al.* 2018).

The majority of TEs in the genomes of *Zymoseptoria* species represents young insertions. TE insertions in these closely related species are species- or strain-specific and one-third comprises copies of highly conserved sequences. Extensive copy-number variation along core chromosomes in the 22 *Z. tritici* isolates strengthens our conclusion that the majority of TEs has spread recently. A study of TE dynamics in worldwide *Z. tritici* populations demonstrated expansions of TE families in isolates of recently founded populations compared to isolates collected close to the center of origin (Oggenfuss *et al.* 2020). More investigations are needed to understand how TE expansions in the closely related sister species occurred, particularly for the more repeat-rich genomes of *Z. passerinii* and *Z. brevis*. It would be interesting to explore if TEs also increasingly accumulate in genomes of *Z. passerinii* and *Z. brevis* isolates outside of the center of origin or if populations of these species show different TE accumulation patterns. Understanding how TEs have accumulated stronger in these genomes requires comparing more *Z. passerinii* and *Z. brevis* isolates.

The high level of young TE insertions in *Zymoseptoria* species contrasts with the high level of RIP mutations found in TE copies. The classical model of TE burst and decay dynamics states that TE proliferation is counterbalanced by genome defense mechanisms with more or less efficacy, which eventually leads to TE elimination (Arkhipova 2018). Here, we observe high level of RIP signatures even on recent TE copies suggesting that most TEs are likely inactive. Based on this, we speculate that the majority of TEs in *Zymoseptoria* were affected by RIP shortly after their insertion. It is worth noting that inactive TEs can still spread in genomes to a certain extent. The duplication of inactive copies may be mediated by recombination that leads to duplication (Bourque *et al.* 2018).

The highly efficient RIP machinery could impede evolutionary innovation in *Zymoseptoria* genomes through TE duplications, and gene duplications are considered a major driver of genome evolution (Galagan and Selker 2004). However, RIP may also have advantageous effects RIP mutations may “leak” into regions encoding protein-coding genes. In this way, RIP was shown to contribute to rapid evolution of effector genes in the fungal pathogen *Leptosphaeria maculans* (Rouxel *et al.* 2011). We found however very few genes exhibiting RIP-like signatures among the genomes of the *Zymoseptoria* species. The details of how RIP occurs in *Zymoseptoria* spp. remain largely unknown. However, one enzyme involved in RIP is the DNA methyltransferase DIM-2. RIP reduction *Z. tritici* isolates outside of the center of origin correlates with a deficiency in DNA methylation and absence of DIM-2 proteins in these isolates (Dhillon *et al.* 2010; Möller *et al.* 2020). For example, there are almost no gene duplications in the genome of the model species *N. crassa* because of extremely high RIP efficacy (Galagan and Selker 2004). In *Z. tritici*, gene duplications are also scarce (Feurtey *et al.* 2020). In this context, host genome defenses against TEs represent a potential fitness cost notably with regard to gene duplication for rapid adaptation (Galagan and Selker 2004).

Moderate transposon activity has the potential to be advantageous for rapid adaptation of fungal plant-pathogens. We propose that a reduction of RIP signatures in repressing transposition in *Z. tritici* isolates outside of the center of origin could represent an advantage for rapid adaptation. The increase of TE insertions in *Z. tritici* could be advantageous during migration to new environments following wheat deployment across the world (Oggenfuss *et al.* 2020). We cannot exclude that the reduction of RIP signatures in the genomes of *Z. tritici* isolates result from a reduction of sexual reproduction frequency. Indeed, as RIP occurs during meiosis, reduction of sexual reproduction cycles can result in a reduction of RIP-mutation cycles and thereby lower amount of RIP-signatures in genomes (Gladyshev 2017). However, previous studies have documented the occurrence of sexual recombination in *Z. tritici* European populations during growing season (Morais *et al.* 2019). In addition, no experimental demonstration has yet to established that RIP signatures are directly linked to sexual reproduction in *Z. tritici*. Indeed, a recent study using experimental evolution in *Z. tritici* has observed RIP-like signatures accumulation during mitosis (Möller *et al.* 2020).
